# The pan-genome as a shared genomic resource: mutual cheating, cooperation and the black queen hypothesis

**DOI:** 10.3389/fmicb.2015.00728

**Published:** 2015-07-21

**Authors:** Matthew S. Fullmer, Shannon M. Soucy, Johann Peter Gogarten

**Affiliations:** ^1^Department of Molecular and Cell Biology, University of ConnecticutStorrs, CT, USA; ^2^Institute for Systems Genomics, University of ConnecticutStorrs, CT, USA

**Keywords:** pan-genome, black queen hypothesis, red queen hypothesis, social cheating, gene transfer

## Cells without complete genomes

Cells have long been recognized as life's building blocks (e.g., Virchow's dictum “*omnis cellula e cellula*,” Virchow, 1860). Specifically, a cell's genome is considered the repository of genetic information that pairs with the cellular machinery to determine the organism's phenotype. Except for rare circumstances, the majority of a genome is passed on from ancestor to descendant, although the acquisition of genes from organisms that are not direct ancestors is recognized to play an important role in evolution (Swithers et al., 2012).

Jeffrey Lawrence, in discussing minimal genome size proposed a meta-cell model (Lawrence, 1999), in which many micelles (small vesicles containing resources, products, and genes) exchange genes frequently. Genes temporarily reside in a micelle and direct the synthesis of compounds important for replication. A micelle only can replicate when all compounds necessary for division have been generated. However, at each point in time only a fraction of the necessary genes are present in an individual micelle. This model relies on gene transfer being so frequent that each of the genes that encode necessary functions visits the individual micelles often enough to allow for sufficient synthesis of the necessary gene products for future micelle divisions. The meta-cell can be considered an organism, whose genome is divided into a network of micelles. Lawrence's meta-cell model is reminiscent of Woese's progenote (Woese, 1998) and Kandler's pre-cell populations (Kandler, 1994) that were postulated to have existed early in evolution before genes coalesced into genomes.

## The pan-genome as a shared genomic resource

For most bacterial and archaeal species different strains contain non-overlapping gene sets. The pan-genome of a taxon or group refers to the sum of all genes that are present in members of the group (Tettelin et al., 2005; Lapierre and Gogarten, 2009). Pan-genomes comprise the core genome, i.e., the genes that are found in all members, and the accessory genome, i.e., genes that are present in only one or a few members of the group. Welch et al. (2002) provided the first illustration that genome content in bacteria changes rapidly. Comparing three *Escherichia coli* strains they found the shared core to be less than 40% of the gene families present in all three genomes. More recently the size of this core was further reduced to only 6% of gene families present in 61 *E. coli* genomes (Lukjancenko et al., 2010). Baumdicker et al. (2012) estimate that the *Prochlorococcus* pan-genome contains about 58,000 genes, whereas the individual genomes encode only about 2000 genes each.

The pan-genome concept was originally developed to explore the fluidity of prokaryotic genomes (Tettelin et al., 2005). Because HGT is more frequent between close relatives (Andam and Gogarten, 2011), the pan-genome may also represent the set of genes that is potentially available via HGT to any member of the group. The function of the pan-genome may then be thought of as a shared resource. This is supported by the observation that genes encoding weakly selected functions are frequently lost from bacterial genomes, when they do not provide selective advantages, only to be re-acquired through HGT, when new conditions provide a selective advantage to carriers (Lawrence and Roth, 1996). The idea of the pan-genome of a population as a shared genomic resource is similar to the description of meta-cells and pre-cell populations. In particular, these concepts have in common that the individual genome of a cell or micelle does not represent a sufficient description of the genomic resources of the population. The following paragraphs discuss some factors that contribute to the large size of pan-genomes.

## The strong black queen hypothesis

**Figure 1:**
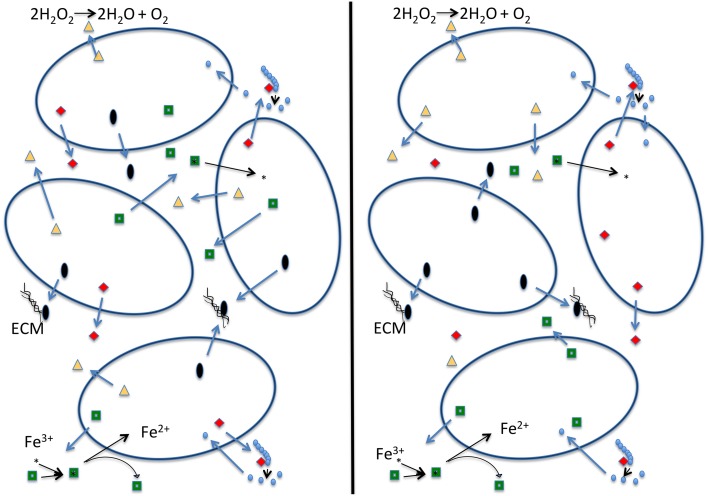
**Schematic depiction of a strong version of the black queen hypothesis (Morris et al., 2012)**. Each cell in the left panel contains all the genes that produce the four depicted common goods. Cells on the right, due to selection to minimize genome size, each produce only one of the common goods, and rely on the presence of other cells to produce the other ones. We consider this a strong version of the black queen hypothesis because it no longer contains exclusive helper strains; rather all individuals in the population described in the right hand panel are simultaneously helpers and beneficiaries. In this example dark open ovals represent individual cells; red diamonds: hydrolases that digest an extracellular polymer (e.g., phosphatase, sialidase, glucanase); green squares: siderophores that allow cells to acquire iron; black ovals: building blocks of the extracellular matrix (ECM) or enzymes that produce and assemble these building blocks; yellow triangles: enzymes that destroy oxygen radicals (e.g., catalase—reaction given; or peroxidase—reaction: ROOR' + electron donor (2 e^−^) + 2H^+^ → ROH + R'OH).

The black queen hypothesis proposed by Morris et al. (2012) is built on the premise of “leaky” common good functions, which cannot be restricted to benefit only the producer. The hypothesis suggests that these functions combined with selection for small genomes may lead to a situation in which these leaky functions are encoded in only a fraction of the genomes comprising the community. Under the black queen hypothesis a cell's evolution can follow one of two pathways (see Figure 1): (1) the cell can retain all genes encoding leaky functions (in the game of hearts, from which the name for the black queen hypothesis derives, this strategy is known as “shooting the moon”). The cost is a large genome maintaining and expressing many genes that are not essential to central metabolism, growth, and reproduction. Consequently, maintaining these genes and expression of extra proteins competes for cellular resources that could be put toward replication and results in a lower growth rate (Dong et al., 1995; Scott et al., 2010; Weiße et al., 2015). The advantage of the “shooting the moon” strategy is that following a population bottleneck all genes encoding leaky functions are available in the genome. These members of a community following this strategy may be thought of as analogous to a keystone species. (2) The cell looses some or all of its leaky functions and increases its growth rate (in hearts, this represents the usual strategy of taking as few point cards as possible). Traditionally this is described as cheating, as the second strategy relies upon other cells in the population for the leaky functions it has lost. If a bottleneck occurs, a single cheating cell is unlikely to survive on its own. A possible outcome of all cells in a population following strategy #2 is that all members of a population cheat on some leaky functions. The members in the population then become mutually dependent on one another (Figure 1). In this scenario there are no keystone members providing all of the leaky functions. For the population to establish itself in a new environment several members of the population are required for the migration to be successful, as no single cell has all the components necessary to sustain itself. We term this the “strong” version of the black queen hypothesis. If all members of a population follow the second strategy, this may under some conditions lead to instability, the tragedy of the commons, and extinction of the population; however, experimental work by Morris et al. (2014) has shown that partitioning of a leaky common-goods function can enable the stable co-existence of two very similar organisms that use the same resources. Additionally, under natural conditions cells do rarely exist in homogeneous mixture (Davey and O'toole, 2000). Cells existing in biofilms or small aggregates are likely to be proximal to cells with which they share recent ancestry, and therefore proximal cells will have the same genotype with respect to shared functions. Drescher et al. (2014) show that *Vibrio cholera* can avoid the public goods dilemma by strengthening relationships between cells of the same genotype through creation of a thick biofilm, thereby providing a local selective advantage to producers of a particular common good in case this good becomes an overall limiting resource. It seems likely that genes encoding common goods are under frequency dependent selection, leading to local feedback loops that contribute to a long-term co-existence of the different types of cheaters.

## Black vs. the red queen

Bacteria are under severe predation by phage (Thurber, 2009). They need to constantly change to evade predation, hence the analogy to the red queen from Lewis Carroll's (Carroll and Gardner, 1999) *Through the Looking-Glass*, who needs to run as fast as she can just to stay in place (Van Valen, 1973). The analysis of phage metagenomes and rank abundance curves indicated that the phage predation follows the *kill the winner* strategy (Hoffmann et al., 2007), where successful strains are targeted more frequently. The surprising long term stability of species composition despite phage predation suggests that cycling between different susceptible target cells occurs within a population and not between populations from different species (Rodriguez-Brito et al., 2010). Consequently, within a population, host genes that encode receptors utilized by phage and virus to enter the cell are expected to turn over quickly, creating within population diversity (Chaturongakul and Ounjai, 2014).

## Random acquisition of genes

Genes are constantly acquired by genomes, and many of the transferred genes do not find a long term home in the recipient genome (Lawrence and Ochman, 1997). Among these genes are parasites (prophages) and selfish genetic elements. Most, but certainly not all (Lobkovsky et al., 2013), of the transferred genes are selectively neutral or nearly neutral to the recipient (Gogarten and Townsend, 2005; Baumdicker et al., 2010; Haegeman and Weitz, 2012). Though these genes may not find long term homes in the genomes they “visit,” selfish genes especially can affect the rates of gene sharing and thus the size of the pan-genome in a population. Furthermore, many selfish elements induce genome rearrangements that can promote the loss and gain of genes, and thus may have a significant impact on the initiation of the loss of leaky functions.

Generation of paralogs may play a role in facilitation of loss of leaky functions. Additional copies increase gene dosage, ameliorating the loss of function in other members of the population by providing more of the common good. However, the pressure to delete genes from genomes is much stronger than to duplicate them (Mira et al., 2001) and an increase in gene transcription can have a similar or greater effect on the overall expression level (Weiße et al., 2015). Regardless of whether the increased production comes from paralogy or regulation it would need to be countered by a greater decrease in production from other common good functions to overcome the cost of increased protein expression.

## Conclusion

Random acquisition of genes and selfish genetic elements, selection by predators, and cheating on common goods, all undoubtedly play a role in generating diversity within populations of bacteria and archaea. The conjecture of the strong black queen hypothesis is that mutual cheating leads to mutual dependencies and therefore cooperation. Under this hypothesis individual cells would be integrated into a meta-organism, whose genome is the pan-genome of the population, similar to Lawrence's meta-cells whose genome is distributed over individual micelles.

The pan-genome of a population as shared genomic resource could explain part of the “genome of Eden” paradox (Doolittle et al., 2003), where estimations of ancestral genomes are far larger and more complex than those of any extant individual genome. Large estimates of archaeal ancestors' genome sizes (Csurös and Miklós, 2009; Wolf et al., 2012) could actually represent the pan-genome of the ancestral population rather than any single cell. If this is the case, the complexity of the progenitor cells in a lineage/population might often be at a similar level of complexity as their extant relatives. We hypothesize the large estimates of progenitor genome size might in part reflect a “strong” black queen scenario where genome variation creates a large pan-genome, but no single cell contains a “keystone genome” with all genes in the population represented. More extensive studies of individual and population genomes, and rates of within population transfer are needed to confirm that master genomes, encoding all the leaky functions needed for survival of the population, can be and often are absent from a population.

If the hypothesis of the population pan-genome as a shared genomic resource is borne out, then the scientific community will need to continue to increase its appreciation for the import of pan- and meta-genomes. Likewise, we may need to more seriously consider populations as the operative units in which genes are selected in rather than exclusively individual organisms. Similar to how Richard Dawkins (1976) advocated thinking of an organism as a collection of generally agreeable, but selfish, genes perhaps we should be thinking of lineages and populations as the collections of genes, i.e., pan-genomes, rather than the individual cells.

### Conflict of interest statement

The authors declare that the research was conducted in the absence of any commercial or financial relationships that could be construed as a potential conflict of interest.
